# The Warning Knock

**Published:** 1872-09

**Authors:** 


### THE WARNING KNOCK.

Not long ago, a lady guest came down to the breakfast-table, and in the course of conversation remarked in a very casual manner, —

"I was knocked up over early this morning."

"How's that?"

"I waked about daylight, and was thinking over the plans of the day, when such a crashing noise was heard at the head-board of the bed, I thought it was a pistol shot; in one instant I was erect in bed, the next on the floor; but there was nothing there."

"Why, Miss Kate, you don't think it was a warning for you?"

"O! no, indeed; but something's going to happen."

And something did happen; for that very night the sun set over the Jersey sand flats one minute later than it did the day before, and what's more, the moon did not make its appearance at the same moment next day.

In a few days after, the excellent lady sickened; and after a brief interval further, I stood by her grave in Greenwood, under the exceedingly impressive offices of the Episcopal Church.

The intelligent reader will see in this a mere coincidence. Cracks and noises in wooden furniture are a frequent result from incessant shrinkage and expansion; it is this which often makes the thunder of the avalanche, the iceberg, and the land slide; bureaus, bedsteads, and tables are liable to these changing conditions from heat to cold, from dryness to moisture, and the reverse. Such occurrences have a very depressing influence on some minds, and in feeble conditions of the body may be the pivot of life and death, of recovery or the grave; and it is well that the reader should have a rational view of such things while in health, especially as the weakening influence of disease impairs the reasoning powers.

